# Mapping Distressing Behaviors in Dementia to Non‐pharmacological Treatments

**DOI:** 10.1002/alz70858_105826

**Published:** 2025-12-25

**Authors:** Aimee R Mooney, Christina S Zonker, Heather Franklin, Emily Tupper, Allison Lindauer

**Affiliations:** ^1^ Oregon Health & Science University, Portland, OR, USA; ^2^ NIA‐Layton Aging & Alzheimer's Disease Research Center, Portland, OR, USA; ^3^ OHSU School of Nursing, Portland, OR, USA

## Abstract

**Background:**

Non‐pharmacological treatments (NPTs) for individuals with dementia and their caregivers have been shown to increase activity participation, maximize independence and reduce caregiver burden. The World Health Organization (WHO) has directed rehabilitation as a core component of dementia care. Dementia family caregivers commonly relate upsetting behaviors, e.g., “refuses to bathe” and “does nothing all day”. This study maps distressing behaviors reported by dementia caregivers onto NPTs.

**Method:**

Distressing behaviors were specified by family caregivers as part of a larger psychoeducational intervention project: Tele‐STELLA, (Support via Technology: Living and Learning with Advancing dementia, NIA R01AG067546). Definitions, treatment components, targets and aims of NPTs were identified and through consensus, condensed into a final set of seven NPTs; coding rules were established. Two researchers mapped a sampling of 98 behaviors onto the NPTs, achieving substantial inter‐rater reliability, with an agreement of 79% and a Cohen's Kappa of 0.68 [95% CI (0.56, 0.80)].

**Result:**

73% of the behaviors were categorized to the NPTs of Cognitive Rehabilitation/Occupational Therapy (43), Expressive/Receptive Language Therapy/Swallowing (16) and Psychological Management (13); 2% of behaviors were coded as ‘No NPT’. Agreement was not reached on 21% of behaviors, which occurred when the distressing behavior was not clearly defined, for example, “gets verbal when things don’t go her way”, indicating further revision of coding rules are necessary.

**Conclusion:**

By categorizing the caregiver‐generated distressing behaviors to relevant NPTs, we highlight the need for referral to rehabilitation services in dementia care. This analysis suggests many dementia caregiver concerns map neatly to rehabilitation services, primarily those focusing on Cognitive Rehabilitation, Occupational Therapy, Speech and Language Treatment, Swallowing, Nutritional Treatment or structured Psychological Therapy. This offers practical information for health care providers when considering referral options to enhance function and reduce burden. NPTs are multi‐tiered, and several might be used to manage behaviors and improve outcomes. Coding refinement and staff re‐training for clarity in behavioral description may improve reliability of characterization or may highlight the need for multidisciplinary intervention.

